# The Hospital Officer's Bookshelf

**Published:** 1912-04-06

**Authors:** 


### THE HOSPITAL OFFICER'S BOOKSHELF.

Outlines of Biology. By P. Chalmers Mitchell, LL.D. and G. P. Mudge, F.Z.S. (London: Methuen and Co., Ltd. Price 6s. net.)

"Chalmers Mitchell" is known to every medical student, since it has for a very long time enjoyed a well-deserved reputation as the text-book for the biological part of the "First Conjoint." The new edition has been ably revised, and contains a large amount of new material which brings it fully up to date. Many new illustrations have been added, and the book still maintains its place as the best elementary manual on the subject with which it deals.

Model Answers to Questions Set by the Medico-Psychological Association. By Hector Macphail. (London: The Scientific Press. Price 2s. net.)

The plan of analysing examination papers for the previous ten years or so, and of building up model answers into an arranged scheme of instruction, is a very old one and one which has made the reputation of many a "crammer." It is objectionable when it leads to a neglect of essentials in the art or science concerned; and indeed to succeed then it necessitates bad examining. But it is not objectionable when the course of instruction thus built up includes every principle of importance and every detail founded on those principles. For in that case it must mean that the whole syllabus of the subject is adequately covered, and that the "crammer" really converts himself into an unusually thorough and painstaking teacher. In respect of this publication, it needs only to say that the latter method is that which the author has adopted; and that his *model answers* constitute in reality an exceptionally well-arranged and clearly expressed compendium of the curriculum which candidates for these examinations have to master. It may therefore be recommended without reserve to those who intend to pass these examinations, and will doubtless prove both valuable and popular.

Hygiene and Public Health. By Sir Arthur Whitelegge, K.C.B., M.D., F.R.C.P., and Sir George Newman, M.D., F.R.S.E. Twelfth Edition, revised throughout. Illustrated. (London: Cassell and Co., Ltd. Pp. 760. Price 8s. 6d.)

The revision of this favourite text-book could not have been a very easy matter for the authors, and we imagine that they must have found some difficulty in preserving one of its most important features—we might even describe it as the most popular feature—namely, its small and handy size, so convenient for the student's pocket. Considering the great variety of subjects which have to be dealt with, ranging from elementary chemistry to sanitary law, it is not a matter for surprise that here and there weak spots might be found by a captious critic. But we may still have no hesitation in saying that as a text-book for the D.P.H. the little red book remains unbeaten. This twelfth edition has been brought well up to date, especially in the chapter on School Hygiene, which includes the memoranda issued by the Local Government Board and the Board of Education. The general plan of the book remains the same, and by this time must be familiar to many hundreds of students; we are glad to note the appearance of a few new and improved illustrations. Whether it is wise to include so much in so small a compass is open to question, but in the 700 odd pages the student will find a relatively enormous amount of information on all those diverse interests which come into the purview of a Medical Officer of Health.

Further Researches into Induced Cell-Reproduction and Cancer. (The McFadden Researches.) By H. C. Ross, M.R.C.S., Eng. (London: John Murray. Pp. 63. Price 3s. 6d. net.)

Continuing the researches the results of which were published about a year ago, Mr. H. C. Ross has now brought forward some further papers written by himself in conjunction with Dr. J. W. Cropper and Mr. E. H. Ross detailing the technique of and the results obtained from additional experiments on induced cell division conducted at the Liverpool School of Tropical Medicine. The experimenters believe that normal cell-reproduction is not merely an intrinsic vital function or "duty" on the part of the cell, but is directly caused by chemical agents. Whether the figures which these workers have induced in cells, and which they interpret as division figures, are really the phenomena of cell division or not is a matter which has been the subject of a considerable amount of criticism. If they are correct, the whole teaching of cytology will need almost revolutionary revision.

First Aid to the Injured and Sick: An Advanced Ambulance Handbook. By F. J. Warwick, B.A., M.B., and A. C. Tunstall, M.D., C.M., F.R.C.S. Seventh Edition, Revised. Bristol: John Wright and Sons, Ltd.)

Ambulance text-books are greatly improved at the present day compared with those of a decade back, and this little volume represents an excellent type of manual. The physiology and anatomy section is brief and compact, yet sufficiently explicit. Bandaging and first-aid treatment is explained in a careful and thoroughly practical fashion, while the exceedingly numerous illustrations are clear and of great assistance. The matter of invalid transport and stretcher-drill is very fully treated, and has been brought up to date in accordance with the new manual of the Royal Army Medical Corps. The illustrations of this section are clear and serve satisfactorily to elucidate the somewhat intricate movements of stretcher-drill. As a condensed yet exhaustive manual for more advanced first-aid students we consider this book of very great merit.

British Red Cross Society First-aid Manual. By James Cantlie, F.R.C.S. (London: Cassell and Co. 1912. Pp. 219. Price 1s. net.)

A short time ago (The Hospital, February 17, 1912) attention was directed in these columns to Mr. Cantlie's advanced manual of training for voluntary aid detachments. In that he broke much fresh ground; but in this first-aid manual he is covering old and well-worn tracks. Briefly, we fail to see that this is any improvement on the St. John Ambulance handbook on the same subject by the same author. It is somewhat larger, because more anatomy has been included, which in itself is a mistake. The subject is still apportioned into five lectures, which are too few for a thorough exposition even of the shorter St. John course. Finally, as is not unnatural, the new book is largely a repetition of the old one, which has long served its purpose very well. The only criticism we would add is that Mr. Cantlie still fails to make clear to first-aid students the fundamental classification of fractures into simple and compound; indeed, his falling short in this respect is more marked in the new manual than it was in the old one.

Essays on Duty and Discipline: A Series of Papers on the Training of Children in Relation to Social and National Welfare. (London: Cassell and Co. Price 3s.)

A movement, supported by a very numerous and distinguished company of well-known people, has been started with a view to counteract the alleged lack of adequate moral training and discipline among British children of the present day. This volume of essays contains forty, mostly very able, articles bearing on the various aspects of the control and education of children and the best means of fitting them to become worthy, in every sense of the word, citizens of our Empire. The contributions in this volume have been collected from eminent men and women representative of many professions and of different, even opposing, sections of thought. The series is well worth perusal, and the movement, of which the Honorary Secretary is Miss Isabel Marris, is one which should command the earnest consideration and practical support of all those who work among children. The healthy and vigorous tone of the essays should appeal to all intelligent parents, and the dissemination of such literature among them is in itself an ample justification for this movement against slackness, sentimentality, and self-indulgence.

A Surgical Treatment of Locomotor Ataxia. By L. N. Denslow, M.D. (London: Baillière, Tindall and Cox. 1912. Pp. 118. Price 3s. 6d. net.)

In this book the author, an American practitioner who seems to have recently settled in London, expounds a hypothesis of the cause of tabes dorsalis, and a line of treatment based upon it. The hypothesis it is not easy to take seriously; it abounds with defective reasoning based upon unproved assumptions; it is quite unphilosophical, and we cannot feel any confidence whatever in its accuracy. The method of treatment based upon the hypothesis may or may not be good: it would be foolish to condemn it offhand because the underlying hypothesis appears to be faulty. The author reports good results from the treatment, and therefore judgment must be suspended until others have given it a trial and proved it valuable. On the assumption that all tabetics have urethral lesions, which precede and indeed cause the tabes, Dr. Denslow dilates the urethra and applies various caustics, etc., to selected parts of it. This treatment, he avers, cures the symptoms of tabes; and it also cures alopecia, acne, and other diseases in non-tabetic persons. The cases quoted sound like miraculous cures; but the author's enthusiasm for his subject makes one doubt a little whether his mind is quite as free from bias as is desirable in a scientific investigator.

The New Physiology in Surgical and General Practice. By A. Rendle Short, M.D., F.R.C.S. (Bristol: John Wright and Sons. Pp. 200. Price 4s. 6d.)

Why the author should go out of his way to describe his very interesting efforts as intended more for the surgeon than for the physician we are at a loss to understand. It seems to us that the usefulness of his book is greater to physicians than to surgeons, for most of the problems discussed have a distinct medical interest. Dr. Rendle Short has certainly gathered together a most instructive series of chapters in which the more recently accepted conclusions arrived at by physiologists and clinicians are discussed in their bearings on modern treatment. It is, as the author points out, a somewhat thankless task to pull down the strongholds of belief, and we doubt if the older generation of practitioners will reform its ways in consequence of the cold water showered on some of its most cherished methods, beloved moreover by the patients as well. But, if slow, progress is sure, and this book will serve a most useful purpose. We heartily recommend it to the attention of general practitioners who have not yet succumbed to routine, and predict that they will find it awaken new interests in every-day clinical work. The chapters on the applied physiology of digestion and absorption and the action (perhaps we should rather say inaction) of cutaneous analgesics are especially useful. Though the author's style is capable of improvement, and there are weak sports here and there, this work is one which deserves recognition by a much larger class than the surgeons and the candidates for higher examinations in physiology.

Recent Methods in the Diagnosis and Treatment of Syphilis. By C. H. Browning, M.D., and Ivy Mackenzie, M.B. (London: Constable and Co., Ltd., 10 Orange Street, Leicester Square, W.C. Price 8s. 6d. net.)

The authors have collaborated with Drs. Cruickshank, Chislett, Gilmour, and Morton to produce an interesting compilation of the newer methods of diagnosis and treatment of syphilis. There is nothing original in the notes which they have written, but the book is a handy reference manual and gives details of the various modifications now employed in the laboratory and clinical diagnoses of the disease. To the general practitioner who has not at hand the elaborate resources of an up-to-date pathological laboratory, a new microscope with the latest additions, and the other important addenda which the pathologists in our hospital laboratories regard almost in the light of trivialities, the serum and blood diagnosis of a disease will always present considerable difficulties. Most of us who are in private practice will prefer to continue diagnosing cases of syphilis on the clinical evidence only than to invoking the elaborate ritual of modern methods. The continuance of such a policy of conservatism, however justifiable at this moment when there is considerable doubt regarding the importance of the Wassermann reaction, and when the pendulum with reference to salvarsan treatment has swung round the other way, is scarcely to be commended in the interests of patients when the question of diagnosis is involved. It is highly essential that in doubtful cases the aid of these newer methods should be called in and the matter settled once and for all. Is it syphilis or is it not? If it is, then the spirochæte of the disease will be discovered by microscopical examination. But it is as well that practitioners should know that there are other spirochætes, closely similar in shape and appearance to the S. pallida, and that these may lead to mistakes in diagnosis just as much as the clinical data alone may cause errors. The authors devote considerable space to the description of the methods they advocate. Their summary of the salvarsan treatment is, on the whole, fair, although it appears to us to err somewhat on the side of partiality. The chapter dealing with this important branch of the subject is prefaced by a preliminary exposition of the pioneering work of German chemists and pathologists in what is called chemotherapy. Details of twenty-three fatalities under the salvarsan treatment are given; the list can be greatly extended by anyone who has followed the history of the treatment in the German clinics. There are no illustrations in the book—a matter to be deplored—and we think that the authors have been wise in confining their attention to the lucid letterpress. Those who want a concise epitome of modern syphilological literature cannot do better than buy this handbook and give it a place on their shelves.

